# Complexity of patients with or without infectious disease consultation in tertiary-care hospitals in Germany

**DOI:** 10.1007/s15010-023-02166-w

**Published:** 2024-01-26

**Authors:** C. Meyer-Schwickerath, C. Weber, D. Hornuss, S. Rieg, F. Hitzenbichler, S. Hagel, J. Ankert, A. Hennigs, J. Glossmann, N. Jung, The German, Clinical Study Group in Infectious Diseases (DESTINi)

**Affiliations:** 1 Department I of Internal Medicine, Division of Infectious Diseases, University of Cologne, Cologne, Germany; 2 Department of Cardiothoracic Surgery, University of Cologne, Cologne, Germany; 3 Faculty of Medicine, Department of Medicine II, Division of Infectious Diseases, Medical Center - University of Freiburg, Freiburg, Germany; 4 Department of Infection Prevention and Infectious Diseases, University Hospital of Regensburg, Regensburg, Germany; 5 Institute for Infectious Diseases and Infection Control, Jena University Hospital, Friedrich Schiller University Jena, Jena, Germany; 6 I. Department of Medicine, Division of Infectious Diseases, University Medical Center Hamburg-Eppendorf, Hamburg, Germany; 7 Center of Integrated Oncology Aachen Bonn Cologne Düsseldorf, University of Cologne, Cologne, Germany

**Keywords:** Infectious disease consultations, Complexity, PCCL, Length of stay, Gender

## Abstract

**Purpose:**

Patients seen by infectious disease (ID) specialists are more complex compared to patients treated by other subspecialities according to Tonelli et al. (2018). However, larger studies on the complexity of patients related to the involvement of ID consultation services are missing.

**Methods:**

Data of patients being treated in 2015 and 2019 in four different German university hospitals was retrospectively collected. Data were collected from the hospitals’ software system and included whether the patients received an ID consultation as well as patient clinical complexity level (PCCL), case mix index (CMI) and length of stay (LOS) as a measurement for the patients’ complexity. Furthermore, a comparison of patients with distinct infectious diseases treated with or without an ID consultation was initiated.

**Results:**

In total, 215.915 patients were included in the study, 3% (*n* = 6311) of those were seen by an ID consultant. Patients receiving ID consultations had a significantly (*p* < 0.05) higher PCCL (median 4 vs. 0), CMI (median 3,8 vs. 1,1) and deviation of the expected mean LOS (median 7 days vs. 0 days) than patients in the control group. No differences among hospitals or between years were observed. Comparing patients with distinct infectious diseases treated with or without an ID consultation, the differences were confirmed throughout the groups.

**Conclusion:**

Patients receiving ID consultations are highly complex, frequently need further treatment after discharge and have a high economic impact. Thus, ID specialists should be clinically trained in a broad spectrum of diseases and treating these complex patients should be sufficiently remunerated.

**Supplementary Information:**

The online version contains supplementary material available at 10.1007/s15010-023-02166-w.

## Background

Due to the aging population, patients treated in the hospital with chronic underlying diseases as well as with complex social situations are increasing in number. These patients are common in all medical fields, especially in internal medicine and the complexity of those patients has been an increasing field of study in the last years [1, 2]. However, patient complexity is not a well-defined term and difficult to measure as more evidence arises that it cannot solely be based on age and multimorbidity [3–6]. According to Shippee et al. [7], complexity in patient care is dynamic and influenced by “personal, social and clinical aspects”. In addition, chronic underlying diseases and use of antipsychotic drugs were factors associated with more complex patients in the primary care setting[8]. In other contexts psychological factors as well as social circumstances were integrated in complexity measurements [9, 10].

Furthermore, economic and controlling parameters measuring the complexity of patients do exist. For example, the patient clinical complexity level (PCCL), case mix index (CMI), length of stay (LoS) as well as deviation from the proposed mean length of stay can be considered as tools to measure patient complexity. The PCCL score is calculated by using the number and severity of secondary diagnoses. With a complex procedure, values between 0 (no comorbidities) and 6 (most severe comorbidities) are calculated [11]. In addition, the German DRG system allocates each DRG a defined number of days as a proposed length of stay for the patients. Thus, the deviation of this proposed mean length of stay is able to tell whether patients stay shorter or longer in the hospital than initially calculated [12].

Tonelli et al. [2] defined patients ‘complexity as a compound of clinical, treatment and organizational factors. Using this model, the authors state that nephrologist treat the most complex patients followed by infectious disease (ID) specialists. Moreover, it has been suggested that in case of infectious diseases patients’ case complications besides clinical and psychosocial challenges play an important role in the perceived complexity of a patient [13]. In addition, Rieg et al. [14] observed in a 1-month cross-sectional survey that infectious disease consultation services are involved in the treatment of highly complex patients as measured by case mix index and inpatient length of stay.

However, larger studies comparing the complexity of patients in terms of economic definitions related to the involvement of infectious disease consulting services are missing so far. In addition, even though the importance of infectious disease specialists in the treatment of patients has been increasingly recognized in Germany over the last years with establishing the specialty “Internal Medicine and Infectious disease” as one major step in the way [15], treatment of infectious disease patients is not well enumerated in the current DRG system used for hospital revenue purposes in Germany [16, 17]. Thus, the objective of the study was to compare the economic complexity of patients with and without involvement of infectious disease consultation services regarding common infections and development over time.

## Materials and methods

### Study design

For this study, data of patients being treated in 2015 and 2019 in four different German university hospitals (A-D) was retrospectively and anonymously collected. Data was collected from the university hospitals’ own controlling software and included age, gender, number of secondary diagnoses, length of stay (LOS), deviation of proposed mean length of stay, case mix index (CMI), patient clinical complexity level (PCCL), type of discharge, intensive care unit (ICU) stay and whether the patient received an infectious disease consultation (IDC) while being treated. Patients younger than 18 years of age as well as patients with a length of stay less than 3 days were excluded from the analysis to avoid bias favoring uncomplicated cases. Further, in case the patient had multiple stays throughout the studied years, only the first stay was included in the analyses. Data of 2019 was chosen, as data from later years would have been altered due to the COVID-19 pandemic. To compare data over time an earlier year was chosen and 2015 was the last year with available data in the billing system of the university hospital of cologne.

For the first part of the study, patients receiving an IDC while being treated in the hospital (IDC group) were compared to patients of the same year not being seen by an infectious disease specialist (non-IDC group) throughout their stay. For the second analysis, patients with distinct infectious diagnoses as one of their main diagnosis were assigned to diseases groups: pneumonia, urogenital infections, soft tissue infections, bone and joint infections, and neurological infections. The allocation to the disease groups was based on certain ICD-10 codes (supplement: Table 2). This was then followed by another comparison of patients receiving an ID consultation to patients not being seen by an ID specialist treated in the same year within the disease group. Patients with cardiovascular infections were not assigned to certain disease groups, as ICD-10 codes for those patients were not distinct enough to differ between infectious and non-infectious causes.

ID consultations in the participating hospitals are in most cases formally requested through the hospital IT service and include a written report and a bedside visit. Minor number of consultations are also provided via telephone as described earlier by Rieg et al. [14]. In addition, there are automatic consultations established for specific diseases such as *Staphylococcus aureus* blood stream infections.

### Statistics

Descriptive statistics were carried out for the comparison of demographic parameters as well as complexity measurements between groups. In addition, Mann–Whitney *U* test and Chi-square test were used to analyze differences in complexity parameters between the groups. To prevent statistical interaction, the comparison of complexity parameters was done individually for each parameter.

To evaluate whether differences in the types of discharge and length of stay could be observed when comparing similar patient groups, adjustment for possible confounding factors between groups was performed by a 1:1 propensity score matching. Matching variables were age, gender and PCCL, as the PCCL score is a validated controlling parameter to measure clinical patient complexity based on the ICD diagnoses of the patient. The statistics were performed using IBM SPSS Statistics (Version 28.01.1.1). Graphs were designed using GraphPad Prism and Office Powerpoint (Version 16.75).

### Ethics

The Ethics committees of the participating university hospitals approved the study (for university hospital of cologne: vote 21-1400).

## Results

### Comparison of IDC group vs. non-IDC group

In total, 215.915 patients were included in the study, 3% (*n* = 6311) of those were seen by an ID consultant while being treated in one of the hospitals. Patients receiving an IDC throughout their stay had a significantly higher number of secondary diagnoses (median 14 vs 5), a higher CMI (median 3, 8 vs 1, 1) and a higher PCCL (median 4 vs 0) than patients in the non-IDC group. Interestingly, age did only differ slightly between groups (median 63 vs 62 years), while the number of female patients was profoundly lower (10% difference) in the IDC group. Further, patients co-treated by an ID consultant stayed longer in the hospital (median 22 vs 7 days) and had a higher deviation of the proposed mean length of stay (median 7 vs 0 days). Looking at the types of discharge, it became obvious that patients in the IDC group had a higher in-hospital mortality (13% vs 2%) and were more often transferred to other clinics (16% vs. 4%) and rehabilitations centers (7% vs 2%), while patients in the non-IDC group were more often discharged to home (Table 1). The seen differences in complexity parameters did not differ between each year and between individual participating hospitals (data not shown).

**Table 1:** Comparison of a) patient characteristics and b) clinical outcome parameters of patients receiving an IDC throughout their stay and control patients

ID consultation	Yes	No	
Number of patients	6311	209,604	
a) Patient characteristics			
Age in years (median with IQR)	63 (51; 74)	62 (46; 74)	*p* < 0.001
Number of female patients (total and percentage)	2345 (37%)	99,390 (47%)	*p* < 0.001
Number of secondary diagnoses (median with IQR)	14 (8; 23)	5 (3; 9)	*p* < 0.001
PCCL (median with IQR)	4 (2; 4)	0 (0; 2)	*p* < 0.001
Case mix index (median with IQR)	3.8 (1.6; 9)	1.1 (0.8; 2.5)	*p* < 0.001
Number of patients treated in ICU (total and percentage)	3327 (53%)	40,732 (19%)	*p* < 0.001
b) Clinical outcome parameters			
Total length of stay in days (median with IQR)	22 (13; 38)	7 (4; 11)	*p* < 0.001
Deviation of mean length of stay (median with IQR)	7 (0; 17)	0 (− 1.8; 3)	*p* < 0.001
In-hospital death (number with percentage)	818 (13%)	4552 (2.2%)	*p* < 0.001
Discharge to home (number with percentage)	3800 (60%	186,320 (89%)	*p* < 0.001
Transfer to other hospital (number with percentage)	1015 (16%)	9314 (4%)	*p* < 0.001
Discharge to rehabilitation (number with percentage)	471 (8%)	4292 (2%)	*p* < 0.001

*ID* infectious disease, *IQR* interquartile range, *PCCL* patient clinical complexity level, *ICU* intensive care unit

### Patients with distinct infectious diseases

For a second analysis, we assigned patients to distinct disease groups (pneumonia, urogenital infections, soft tissue infections, bone and joint infections and neurological infections) when one of the infectious diseases was coded as a main diagnosis. Numbers of patients receiving IDCs in the different disease groups differed clearly, as only 3% (*n* = 44) of patients with urogenital infections and 6% (*n* = 146) with pneumonia were seen by an ID specialist while, respectively, over 30% of patients with bone and joint infections or neurological infections were in need of an IDC throughout their treatment. There were again less female patients in the IDC groups than in the non-IDC groups with the same underlying diseases. However, the difference in the proportion of female patients between IDC patients and non-IDC patients varied from 3% for bone and joint infections to 15% for urogenital infections. Further, patients with soft tissue infections, bone and joint infections as well as neurological infections and in need of an IDC were older than the control groups, while patients with pneumonia and co-treatment by an ID specialist were significantly younger than their counterparts in the non-IDC group.

When looking at complexity parameters, it became obvious that patients with distinct infectious diseases and in need of an IDC had again a higher CMI, higher number of secondary diagnoses and a higher PCCL than patients with the same diagnosis in the non-IDC group. Further, all patients receiving an IDC and regardless of the underlying disease had a longer in-hospital stay than patients not in need of an IDC. Of note, the deviation of the proposed mean length of stay was also higher for patients receiving an IDC throughout the disease groups with one exception for soft tissue infections (data shown in supplement Table 1).

### Evaluation of clinical outcome parameters

In a second step, a propensity score matching was performed to evaluate whether differences in the types of discharge and length of stay could be observed between patients in need of an IDC and the control group when adjusted to age, gender and PCCL as a complexity measurement parameter. As shown in Table 2, even after the matching, patients receiving an IDC had a higher total length of stay (median 22 vs 9 days) and a higher deviation of the mean length of stay (median 6.8 vs 0 days). Further, patients receiving an IDC still had a significantly higher in-hospital mortality (12.9% vs 4.7%) and were more often transferred to another hospital or a rehabilitation clinic.

**Table 2:** Comparison of patients receiving an IDC throughout their stay matched by a propensity score matching to control patients based on gender, age and PCCL

ID consultation	Yes	No	
Number of patients	6256	6256	
Total length of stay in days (median with IQR)	22 (13; 38)	9 (5; 16)	*p* < 0.001
Deviation of mean length of stay (median with IQR)	6.8 (0; 17)	0 (− 3; 3)	*p* < 0.001
In-hospital death (total and percentage)	806 (12.9%)	295 (4.7%)	*p* < 0.001
Discharge to home (total and percentage)	3774 (60.3%)	5313 (84.9%)	*p* < 0.001
Transfer to other clinic (total and percentage)	1009 (16.1%)	327 (5.2%)	*p* < 0.001
Transfer to rehabilitation (total and percentage)	461 (7.4%)	189 (3. = 5)	*p* < 0.001

*ID* infectious disease, *IQR* interquartile range

## Discussion

Looking at our data and the above-mentioned aim of the study, we can strike out three important observations.

1. Patients receiving an IDC are highly complex when looking at economic complexity measurements, have a higher in-hospital mortality and are treated in hospital for a longer period. This observation could be confirmed when matching patients in need of IDCs to controls based on age, gender and PCCL.
2. The observed trends in the differences between the IDC and the non-IDC group are consistent over the years 2015 and 2019.
3. Patients receiving an IDC are mostly male, reflected in a distinct higher male proportion in the IDC group compared to the non-IDC group. This fact is reproduced when looking at the individual disease groups.

Our study points out that patients seen by ID specialists in the form of ID consultations are highly complex when looking at economic complexity measurement parameters. They have a higher number of comorbidities, a higher CMI as well as PCCL, have a longer length of stay, have a higher in-hospital mortality and are transferred to rehabilitation centers more frequently than patients not seen by ID specialists. These results are in line with previously published work stating that infectious disease patients are highly complex using different methods measuring complexity [10, 13, 14, 18–20]. However, even though Grace et al. [20] pointed out the complexity of patients receiving ID consultations, our study is the first one comparing the complexity of patients receiving ID consultations to controls. To our knowledge, our study is the first one showing data on how complex patients receiving ID consultations are in comparison to controls. Eventhough this aspect seems to be predictable from a clinical point of view, questions asked to ID consultants are heterogenous and vary in attention to detail [14], and thus our paper is the first one confirming the assumption that IDCs are asked for highly complex patients. Further, this study is the first pointing out the reproducibility of these seen observations over time in the participating study sites, as previous work mostly looked at shorter time periods.

After all, our results strengthen the risen claim that physicians treating these complex patients need to have an in-depth training in infectious diseases as well as internal medicine, as many of these patients have a high number of secondary diagnoses needing to be taken into consideration when recommending treatment regimens and diagnostics [16, 21]. In addition, even though not always visible in clinical practice, one can assume that the ID consultations service is called especially to the critical and complex patients, suggesting a working selection mechanism in the participating study hospitals.

As stated above, even after matching of patients receiving ID consultations to controls based on age, gender and PCCL, we could observe among other differences a higher number of secondary diagnoses, a longer in-hospital stay as well as a higher mortality in the group receiving ID consultations. For us, this reflects the fact that ID consultations are asked for patients with acute and highly severe infectious diseases or complications. However, as one can also observe a higher deviation of the mean length of stay in the group receiving ID consultations, one can argue that the severeness of these infectious diseases and complications are not displayed in the German billing data. Saying that specific DRGs and codes of operations and procedures for these severe infectious diseases and complications are missing in Germany. These results again stress the claim for a sufficient reimbursement for ID-related procedures and especially ID consultations, as these are currently not displayed in the German hospital payment system [14, 17].

Further, as stated above, we overserved a profound higher male proportion in the group receiving an IDC even when looking at the distinct infectious disease groups. This observation is in line with previous published work stating that male patients are more prone to infections [22]. In addition, there is data that male patients are at greater risk of a more severe disease course [23–25]. As we stated above, our results underline that the IDC service is called to treat complex patients with a severe disease progression. Thus, our results underline observed epidemiological differences with regard to susceptibility of male and female patients to infectious diseases as well as with regard to the differences in the disease course.

To our knowledge, our study is the first to compare patient complexity with regard to involvement of ID consultation service with these high patient numbers. Nevertheless, our study has some limitations. First, interactions between outcome parameters cannot be excluded from our study. The CMI for example has overlapping aspects with the PCCL and the number of secondary diagnosis. However, as complexity is difficult to assess, clear definitions of which parameters to use are difficult to find [3, 4]. As the used parameters define complexity from a billing data point of view, it is one way to express complexity in figures. Due to the retrospective design as well as the type of data used for the analysis, several questions cannot be answered. We do not have information on the type of secondary diagnoses as well as on the prescribed medications, social factors or psychological implications used in other measurements of patient complexity [10, 13]. This further data cannot be obtained subsequently, as the initial data was collected anonymously without possibility to relate to individual patients. In addition, as mentioned above, no data on individual patient’s cost were obtained due to local ethical reasons. These data, however, could give a better insight into the enumeration of ID consulting services and thus should be part of further investigations. Further, we cannot state whether the recommendations made in an ID consultation reduce the individual patient morbidity and mortality. There is data available stating that involvement of ID specialists in certain infectious diseases such as *Staphylococcus aureus* and *Candida* bloodstream infections as well as endocarditis improves patient outcome [26–30], but larger studies looking at different infectious diseases are missing so far. A further prospective study to answer this among other questions was initiated by the study group and is currently recruiting patients (DRKS00027299).

### Supplementary Information

Below is the link to the electronic supplementary material.

Supplementary file1 (DOCX 26 KB)

## Data Availability

The data generated and analyzed during the study are available from the corresponding authors on request.

## Code availability

The code for data analysis is available from the corresponding authors on request.
