# Supplementary material for: Complexity of patients with or without infectious disease consultation in tertiary-care hospitals in Germany
Source: Infection. 2024 Jan 26;52(2):577–82. doi: 10.1007/s15010-023-02166-w (PMC10955003; doi:10.1007/s15010-023-02166-w)
Supplement: Supplementary file 1 — Supplementary file1 (DOCX 26 KB) [file 15010_2023_2166_MOESM1_ESM.docx]

**Supplement tables**

|  | **pneumonia** | | | **urogenital infections** | | | **soft tissue infections** | | | **bone and joint infections** | | | **neurological Infections** | | |
| --- | --- | --- | --- | --- | --- | --- | --- | --- | --- | --- | --- | --- | --- | --- | --- |
|  |  |  |  |  |  |  |  |  |  |  |  |  |  |  |  |
| **ID consultation** | yes | no |  | yes | no |  | yes | no |  | yes | no |  | yes | no |  |
| **number of patients** | 146 | 2103 |  | 44 | 1429 |  | 199 | 1485 |  | 198 | 398 |  | 181 | 410 |  |
| **age in years**  (median with IQR) | 66,5  (56; 77) | 72  (59; 81) | p = 0,008 | 63  (52; 73) | 66  (46; 77) | p = 0,548 | 63  (47; 73) | 57  (44; 72) | p = 0,221 | 69,5  (60; 78) | 64  (52; 74) | p < 0,001 | 57  (49; 71) | 45  (31; 58,3) | p < 0,001 |
| **Number of female patients**  (total with percentage) | 37  (25,3%) | 828  (39,4%) | p < 0,001 | 18  (40,9%) | 798  (55,8%) | p = 0,050 | 69  (34,7%) | 641  (43,2%) | p = 0,023 | 74  (37,4%) | 162  (40,7%) | p = 0,434 | 77  (42,5%) | 223  (54,4%) | p = 0,008 |
| **number of secondary diagnoses**  (median with IQR) | 12  (7,8; 20) | 9  (5; 13) | p < 0,001 | 14,5  (6; 22,8) | 6  (3; 11) | p < 0,001 | 12  (7; 18) | 5  (2; 8) | p < 0,001 | 11  (7; 17) | 7  (4; 11) | p < 0,001 | 9  (5; 16) | 4  (2; 6,3) | p < 0,001 |
| **total length of stay**  (median with IQR) | 17  (10; 27,3) | 8  (6; 12) | p < 0,001 | 17  (7,2; 33,8) | 6  (4; 8) | p < 0,001 | so | 7  (5; 10) | p < 0,001 | 26  (17; 39) | 12  (7; 20) | p < 0,001 | 17  (13; 31) | 8  (5; 13,3) | p < 0,001 |
| **deviation of mean length of stay**  (median with IQR) | 10  (4,9; 21) | 4  (-0,9; 9) | p < 0,001 | 10,5  (3,2; 31,2) | 2,2  (-0,2; 5) | p < 0,001 | 2  (-3; 10,5) | 3  (0,1; 7) | p = 0,586 | 11  (3; 25) | 6  (0; 16) | p = 0,015 | 8  (3; 19,9) | 3  (-2,5; 8,2) | p < 0,001 |
| **PCCL**  (median with IQR) | 3  (2; 4) | 3  (1; 3) | p < 0,001 | 3  (1; 4) | 1  (0; 3) | p = 0,005 | 3  (-3; 11) | 0  (0; 2) | p < 0,001 | 3  (1; 4) | 2  (1; 4) | p = 0,03 | 3  (1; 4) | 0  (0; 2) | p < 0,001 |
| **Case Mix Index**  (median with IQR) | 1,7  (0,9; 4) | 0,8  (0,8; 1,4) | p < 0,001 | 1,2  (0,6; 3,2) | 0,6  (0,5; 0,8) | p < 0,001 | 3,9  (0,9; 7,1) | 0,6  (0,6; 0,7) | p < 0,001 | 3,7  (2,3; 5,4) | 2,4  (1,4; 3,6) | p < 0,001 | 2,8  (1,5; 5,4) | 1,3  (1,3; 1,4) | p < 0,001 |
| **n. of patients treated on the ICU**  (total with percentage) | 61  (41,8%) | 442  (21%) | p < 0,001 | 13  (29,5%) | 51  (3,6%) | p < 0,001 | 115  (57,8%) | 50  (3,4%) | p < 0,001 | 75  (37,9%) | 85  (21,4%) | p < 0,001 | 98  (54,1) | 73  (17,8%) | p < 0,001 |
| **In-hospital death**  (total and percentage) | 30  (20,5%) | 164  (7,8%) | p < 0,001 | 2  (4,5%) | 11  (0,8%) | p = 0,006 | 16  (8%) | 10  (0,7%) | p < 0,001 | 6  (3%) | 7  (1,8%) | p < 0,001 | 6  (3,3%) | 5  (1,2%) | p < 0,001 |
| **discharge to home**  (total and percentage) | 99  (67,8%) | 1629  (77,5%) | p < 0,001 | 34  (77,3%) | 1301  (91%) | p = 0,006 | 99  (49,7%) | 1394  (93,9%) | p < 0,001 | 137  (69,2%) | 333  (83,7%) | p < 0,001 | 122  (67,4%) | 353  (86,1%) | p < 0,001 |
| **transfer to other clinic**  (total and percentage) | 6  (4,1%) | 128  (6,1%) | p < 0,001 | 2  (4,5%) | 41  (2,9%) | p = 0,006 | 71  (35,7%) | 34  (2,3%) | p < 0,001 | 33  (16,7%) | 21  (5,3%) | p < 0,001 | 36  (19,9) | 18  (4,4%) | p < 0,001 |
| **transfer to rehabilitation**  (total and percentage) | 3  2,15) | 5  (0,2%) | p < 0,001 | 0 | 4  (0,3%) | p = 0,006 | 9  (4,5%) | 3  (0,2%) | p < 0,001 | 12  (6,1%) | 19  (4,8%) | p < 0,001 | 17  (9,4%) | 23  (5,6%) | p < 0,001 |

**Supplement table 1** Comparison of Patients with distinct infectious diseases as their main diagnosis receiving an IDC throughout their stay in comparison to control patients with the same underlying disease. ID = Infectious Disease, IQR = Interquartile Range, PCCL = Patient Clinical Complexity Level, n.= number, ICU = Intensive Care Unit

| **Disease Group** | **ICD Codes** |
| --- | --- |
| pneumonia | J10.0  J11.0  J12  J13  J14  J15  J16  J17*  J18  J69.0 |
| urogenital infections | N10  N11  N12  N13.6  N15.1  N16.0*  N30.0  N30.8  N33*  N39.0 |
| soft tissue infections | A46  L00  L01  L02  L03  L08  M72.6 |
| bone and joint infections | M46.2-5  M86.0-9  T84.5  T84.6  T84.7 |
| neurological infections | G00  G01*  G02*  G04.0/2/8/9  G05.0*  G05.1*  G05.2*  G06  G07*  G08  A87  T85.72 |

**Supplement table 2** ICD Codes used for allocation of patients to distinct disease groups.
